# Primary Small Bowel GIST Presenting as a Life-Threatening Emergency: A Report of Two Cases

**DOI:** 10.1155/2017/1814254

**Published:** 2017-03-28

**Authors:** Safi Khuri, Hayim Gilshtein, Abd-alkarim Darawshy, Hany Bahouth, Yoram Kluger

**Affiliations:** ^1^Department of General Surgery, Rambam Health Campus, Haifa, Israel; ^2^Colorectal Surgery Unit, Rambam Health Campus, Haifa, Israel; ^3^Trauma and Acute Care Surgery Unit, Rambam Health Campus, Haifa, Israel; ^4^Surgical Oncology Unit, Rambam Health Campus, Haifa, Israel

## Abstract

Gastrointestinal stromal tumor (GIST) is a rare stromal neoplasm, which represents the most common mesenchymal tumor of the gastrointestinal tract. It is characterized by indolent clinical symptoms, although it can present as a life-threatening emergency. Herein, we present two cases of primary small bowel GIST treated at our department. A 68-year-old female patient presented to our emergency department with a diffuse abdominal pain of acute onset. Imaging studies revealed a mass at proximal jejunum, with a nearby free air and fluid. At surgery, a mass of 9 cm was found at proximal jejunum, 3 cm distal to the treitz ligament, with perforation on the lateral wall of the mass. En bloc resection was performed. Pathology report was positive for gastrointestinal stromal tumor. A 70-year-old male patient presented to our emergency department with 3 days of dark tarry stool and few hours of hematochezia. Computed tomography angiography revealed a mass at the pelvis, with calcifications, attached to the distal ileum, with intraluminal blush of intravenous iodine. At surgery, a mass of 8 cm at the distal ileum was found. Resection of the mass along with a 20 cm of ileum was completed. Histopathology report was positive for malignant gastrointestinal stromal tumor.

## 1. Introduction

Gastrointestinal stromal tumor (GIST), first described by Mazur and Clark (1983) [1], is the most common mesenchymal neoplasm of the GI tract; however it accounts for less than 1% of all GI tumors [2]. It originates from the interstitial cells of Cajal, which are part of the autonomic nervous system of the intestine [3]. The majority of the lesions are benign with a possibility of 20–30% for malignancy [4].

GISTs arise usually from the muscularis mucosa or propria layers and mostly have an endophytic pattern of growth.

The estimated frequency of GIST tumors is 10–20/1 million population [4, 5], and it occurs in patients at the sixth decade of life and can arise anywhere in the GI tract from the esophagus to the rectum. GISTs (mainly tumors larger than 4 cm) may present as abdominal emergencies, including GI hemorrhage, usually due to pressure necrosis and ulceration of the overlying mucosa, intestinal obstruction, or perforation. Perforations are more common for GISTs of the small bowel compared to other anatomical sites [6].

We describe two emergency cases of small bowel GIST treated at our department.

## 2. Case  1

A 69-year-old female patient presented to our emergency department complaining of diffuse abdominal pain. The pain lasted for 4 hours, started abruptly, and was described as sharp, diffuse, and being without radiation. She also suffered from nausea, recurrent vomiting, and reduced appetite. Past medical history included hypertension and recurrent urinary tract infections.

**Figure 1:**
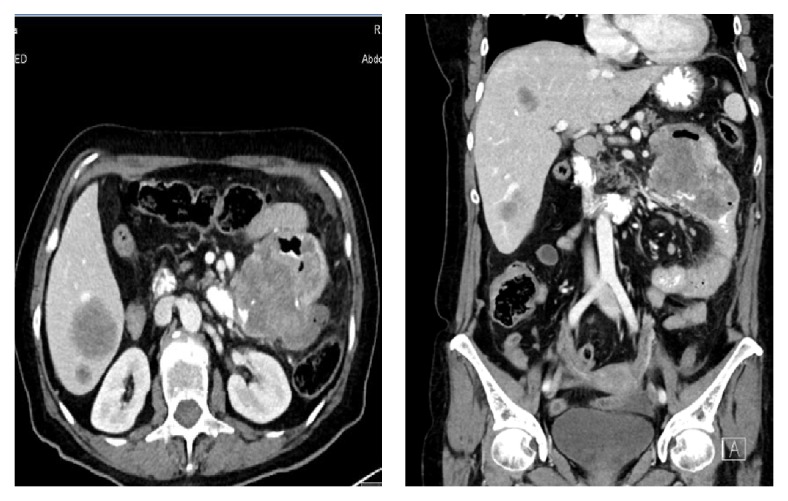
Axial and coronal CT scan showing a mass at proximal jejunum, with nearby free air and liver metastasis.

On physical examination upon her admission, the patient's vital signs showed fever of 39.3 c and tachycardia of 110/min. An abdominal examination revealed diffuse tenderness with guarding. No abdominal mass was palpated. Digital rectal examination was normal. Complete blood count showed increased white blood cells of 14000, with 10% bands. Liver and kidney function tests were within normal limits. An upright abdominal X-ray and chest X-ray were normal. A computed tomography scan revealed a hypodensic mass of 9.5*∗*6.4 cm at proximal jejunum leading to narrowing of the lumen, with a nearby free air and fluid, small amount of fluid at the pelvis, and multiple hypodensic masses at right liver (Figure 1). She was admitted with a diagnosis of abdominal sepsis.

**Figure 2:**
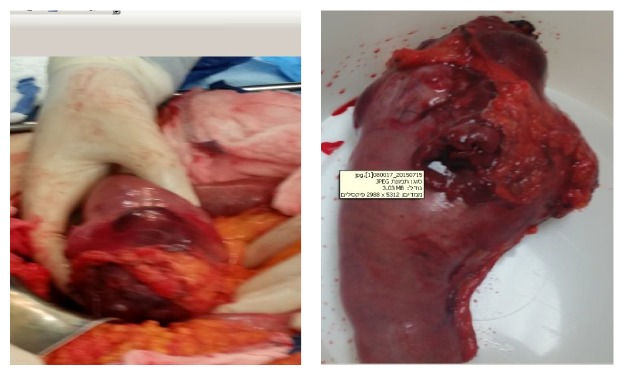
At laparotomy: mass at proximal jejunum with blowout perforation of 1 cm on the lateral wall of the mass.

Due to these findings, the patient underwent an exploratory laparotomy, during which a mass of 9 cm, located 3 cm distal to treitz ligament, was found, with a blowout perforation of 1 cm diameter at the lateral wall along with a large amount of purulent fluid (Figure 2). En bloc resection of the mass along with a 15 cm of proximal jejunum and fourth part of the duodenum was done, and a primary side-to-side anastomosis between the jejunum and second part of the duodenum was performed. A wound protector during specimen extraction was used. Multiple palpable but not visible masses were detected at the right lobe of the liver. Inspection of the rest of the GI tract and abdominal cavity did not reveal associated abnormalities. Her postoperative course was uneventful, and the patient was discharged home on day eight.

The histopathological examination showed a GIST of 10 cm length, made of spindle and epithelioid cells and invading the mucosa. Mitoses were in 5 per 50 high power fields. Immunohistochemically, the tumor cells were positive for CD 117 and DOG-1 and negative for CD34, Desmin, chromogranin, SOX1, HMB45, and S100. These findings are compatible with high risk for recurrence. Postoperatively, the patient was offered but declined adjuvant treatment with imatinib. During two years of postoperative follow-up the patient is alive but declined the planned observation.

## 3. Case  2

A 70-year-old male patient presented to our emergency department due to dark tarry stool of 3 days with a change towards dark red stool in the last hours prior to his admission. He had also general weakness, dizziness, and palpitations. His past medical history included epilepsy.

**Figure 3:**
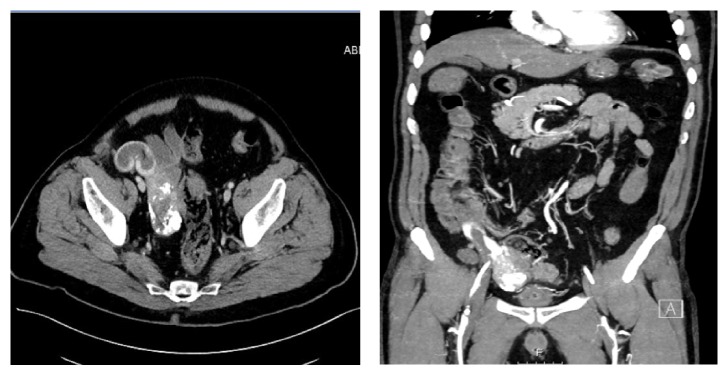
Axial and coronal CT angiography of the abdomen showing a calcified mass at the right pelvis, attached to the distal ileum with intraluminal contrast blush.

On his admission, his vital signs revealed tachycardia of 126/min and blood pressure of 100/60. On abdominal examination, the abdomen was soft and not tender. No abdominal mass was palpated. Digital rectal exam revealed cherry red stool mixed with blood clots. Complete blood count showed hemoglobin level of 6.5 g/dL. Coagulation tests and liver and kidney function test were within normal limits. A computed tomography angiography revealed a lobular mass of 8 cm diameter, with calcifications at the pelvis, attached to the distal ileum, with intraluminal blush of contrast material (Figure 3). He was admitted with a hemorrhagic shock due to gastrointestinal bleeding.

**Figure 4:**
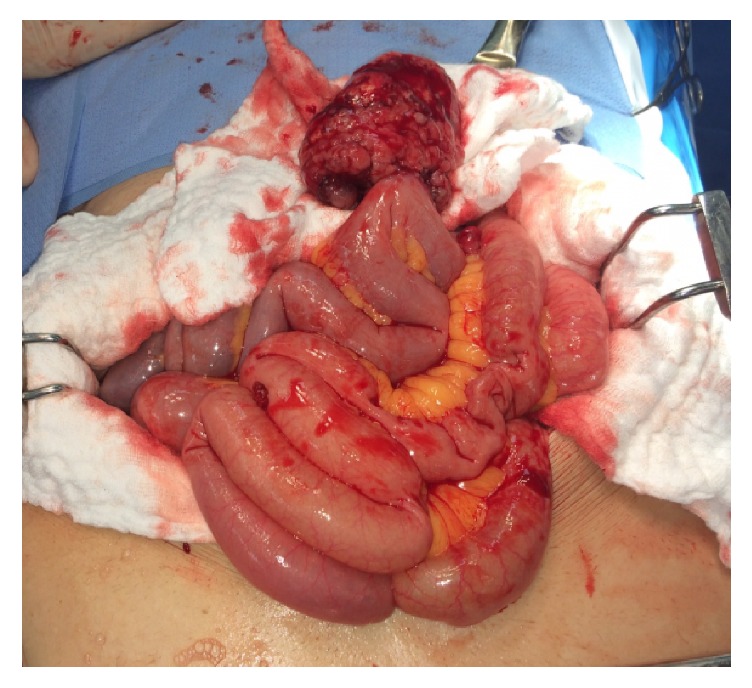
At laparotomy, a hypervascular calcified mass of 8 cm arising from the distal ileum, with ongoing intraluminal bleeding.

Due to these findings, the patient underwent an emergency laparotomy. At surgery, a hypervascular mass of 8 cm diameter at the right pelvis was found. The mass was partly calcified and seemed to rise from the distal ileum (Figure 4). Additional findings were intraluminal blood in the small and large bowel with multiple small masses along the small bowel, mesentery and sigmoid colon. En bloc resection of the mass along with a 20 cm of ileum and primary side-to-side anastomosis was performed. Wound protector was used during specimen extraction as is paramount in these cases. Multiple biopsies were taken from the different masses. His postoperative course was uneventful, and the patient was discharged home on day 8.

The histopathological exam revealed a GIST, epithelioid type of 9 cm diameter, arising in the muscularis propria and extending to the pericolonic adventitia, reaching the serosa. 25 mitotic figures per 50 high power fields were counted, corresponding to tumor risk group 6A.

Immunohistochemically, the tumor cells were strongly positive for CD117 (C-KIT) and DOG-1. Five peri-intestinal lymph nodes were extensively infiltrated by metastatic GIST. Biopsies from the other masses were also positive for malignant epithelioid GIST. Postoperatively, the patient was treated with adjuvant imatinib. On follow-up he is doing well and continues with his daily activities.

## 4. Discussion

GIST tumors are uncommon tumors, and high index of suspicion is warranted for diagnosis [3, 4, 7]. None of the diagnostic procedures, including computer tomography, ultrasound, barium examination, angiography, and magnetic resonance imaging, has a 100% certainty for diagnosis, and preoperative fine needle aspiration is not indicated due to the risk of tumor rupture and intraperitoneal seeding [4, 5, 8]. However, some recent studies have showed the importance of endoscopic ultrasound guided fine needle aspiration, with reported accuracy of 89% [9].

A prognostic classification, proposed by Fletcher et al, depends on tumor size and mitotic counts, classifying GISTs into very low, low, intermediate, and high risk for malignancy (Table 1) [10]. There is slight male predominance [7]. These tumors are mainly sporadic [3], although familial forms with autosomal dominant inheritance have been documented [3, 7].

GIST usually spreads by direct extension to adjacent structures and hematogenously to the liver, lung, and bone. Lymphatic metastasis is unusual [11]. GIST tumors have variable markers, including C-KIT (CD 117), DOG-1, CD 34, SMA, S100, and Desmin, of which, they are almost always positive for C-KIT and DOG 1 [3, 5, 12–14].

The most common site for GIST is the stomach (60–70%), followed by the small bowel (25–35%) [5]. GISTs involving the esophagus, appendix, colon, and rectum are rare, and tumors arising from the omentum, mesentery, or retroperitoneum have been documented; but most of these were found to be metastatic from gastric or intestinal primaries [7].

Jejunal GISTs, which comprise 10% of all GISTs [3], are usually symptomatic and patients suffer from abdominal pain and early satiety. They may also have symptoms secondary to obstruction or hemorrhage. Perforation with acute diffuse peritonitis is rare [4, 15, 16].

Due to the nonspecific symptoms and signs, it is difficult to diagnose jejunal GIST preoperatively. Although specific signs and symptoms are absent [17], most GISTs (70%) are symptomatic, mainly presenting with vague abdominal pain [5]. Other symptoms include nausea, vomiting, early satiety, and abdominal fullness. The remaining (30%) are asymptomatic and diagnosed incidentally. These latter tumors are usually small sized tumors (<2 cm) [3, 5, 12].

Although computer tomography is a viable imaging modality for patients suspected of having intra-abdominal GIST, magnetic resonance imaging modality provides a more accurate preoperative picture [18].

Prognostic factors include anatomic location of the primary tumor, age at presentation, histomorphology, molecular genetics, and immunohistochemistry, of which tumor size is the most important [19].

The treatment of choice is complete surgical resection [11], whenever possible, with 5-year survival of 48–65% [4]. GIST tumors have poor response to chemotherapy or radiotherapy, which is usually used in cases of hemorrhage or for analgesic purposes [5, 12, 20].

Reviewing the current literature revealed only twenty-two cases of acute abdomen with diffuse or localized peritonitis caused by spontaneous perforation of small bowel GIST. Two other cases were reported recently, without mentioning patient's characteristics.

Of these twenty-two cases, 17 patients were males, 5 patients were females, and patients' age ranged from 22 up to 82 years. 15 cases reported the jejunum as the primary site of GIST perforation (1 case report published in different journals) during the years 2006–2012. 4 cases involved the ileum and the other cases were not localized to a specific portion of the small bowel. 19 patients had a primary localized single GIST, 2 patients had a multifocal small intestine GIST, and one patient had hepatic metastasis. All patients underwent an operation with small bowel resection and 61% were treated by postoperative imatinib [21].

We report the second case of spontaneous small intestine GIST perforation with hepatic metastasis at presentation in asymptomatic female patient presented with an acute abdomen due to a perforated jejunal GIST. The diagnosis was made by computed tomography. The primary tumor was resectable.

GISTs have a hemorrhagic potential due to ulceration of the mucosa, although it rarely presents as hemorrhagic shock. Reports showed a high incidence of bleeding, especially for duodenal GIST (87%) and small bowel GIST (64%). Other sites of gastrointestinal GIST, such as stomach, rectum, and colon have a lower incidence of bleeding (<45%).

## 5. Conclusion

Small bowel GIST is an uncommon tumor, with a spontaneous perforation and life-threatening hemorrhage being a rare initial presentation. In order to make the correct diagnosis a high index of suspicion is mandatory, combined with appropriate imaging modalities such as CT scan or MRI of the abdominal cavity.

The mainstay treatment for these emergent presentations of small bowel GIST is resection with a favorable clinical result achievable when the diagnosis is made in a timely manner.

## Figures and Tables

**Table 1:** Fletcher et al. prognostic classification.

	Size (largest dimension)	Mitotic count
Very low risk	<2 cm	<5/50 HPF
Low risk	2–5 cm	<5/50 HPF
Intermediate risk	<5 cm	6–10/50 HPF
	5–10 cm	<5/50 HPF
High risk	>5 cm	>5/50 HPF
	>10 cm	Any mitotic rate
